# An Ribonuclease T2 Family Protein Modulates *Acinetobacter baumannii* Abiotic Surface Colonization

**DOI:** 10.1371/journal.pone.0085729

**Published:** 2014-01-28

**Authors:** Anna C. Jacobs, Catlyn E. Blanchard, Seana C. Catherman, Paul M. Dunman, Yoshihiko Murata

**Affiliations:** 1 Department of Microbiology and Immunology, University of Rochester School of Medicine and Dentistry, Rochester, New York, United States of America; 2 Department of Medicine, Division of Infectious Diseases, University of Rochester School of Medicine and Dentistry, Rochester, New York, United States of America; Loyola University Medical Center, United States of America

## Abstract

*Acinetobacter baumannii* is an emerging bacterial pathogen of considerable medical concern. The organism's transmission and ability to cause disease has been associated with its propensity to colonize and form biofilms on abiotic surfaces in health care settings. To better understand the genetic determinants that affect biomaterial attachment, we performed a transposon mutagenesis analysis of abiotic surface-colonization using *A. baumannii* strain 98-37-09. Disruption of an RNase T2 family gene was found to limit the organism's ability to colonize polystyrene, polypropylene, glass, and stainless steel surfaces. DNA microarray analyses revealed that in comparison to wild type and complemented cells, the RNase T2 family mutant exhibited reduced expression of 29 genes, 15 of which are predicted to be associated with bacterial attachment and surface-associated motility. Motility assays confirmed that RNase T2 mutant displays a severe motility defect. Taken together, our results indicate that the RNase T2 family protein identified in this study is a positive regulator of *A. baumannii*'s ability to colonize inanimate surfaces and motility. Moreover, the enzyme may be an effective target for the intervention of biomaterial colonization, and consequently limit the organism's transmission within the hospital setting.

## Introduction

*Acinetobacter baumannii* has recently emerged as an important nosocomial pathogen that can cause a variety of infections, ranging in severity from minor skin and soft tissue infections to ventilator-associated pneumonia and bacteremia, the latter of which is associated with mortality rates as high as 69% [1], [2]. The success of *A. baumannii* as a human pathogen can, in part, be attributed to its ability to resist most antibiotic treatment regimens. Indeed, the Infectious Diseases Society of America (IDSA) has designated *A. baumannii* as one of the six ESKAPE bacterial pathogens (*Enterococcus faecium*, *Staphylococcus aureus*, *Klebsiella pneumoniae*, *Acinetobacter baumannii*, *Pseudomonas aeruginosa*, and *Enterobacter sp.*) of immediate U.S. health care concern that collectively cause the majority of nosocomial infections and can escape the therapeutic activity of currently available antibiotics [3].

The prevalence of *A. baumannii* disease has been linked to its ability to colonize and persist on abiotic surfaces common to health care settings, thereby providing reservoirs for transmission and infection. Indeed, the organism can readily colonize inanimate surfaces, such as hospital bed rails and ventilator machines, and remains viable on these surfaces for extended periods of time in a physiological state that is resistant to desiccation and disinfectants [4]–[8]. The subsequent direct transmission (or indirect via health care workers) of *A. baumannii* to susceptible patients has been associated with outbreaks of ventilator-associated pneumonia, bacteremia, and wound infections [9]–[12].

The organism's persistence on abiotic surfaces is thought to be mediated by its ability to form robust biofilms on inanimate materials [13]. Accordingly, a number of investigators have begun to define the molecular components that mediate *A. baumannii* biofilm formation and maintenance. Actis and colleagues found that the CsuA/BABCDE chaperone-usher system is required for pili formation and surface attachment during biofilm formation on polystyrene surfaces [14]. The extracellular polysaccharide poly-β-(1,6)-N-acetylglucosamine (PNAG) is hypothesized to subsequently serve as an intracellular adhesion among biofilm-associated *A. baumannii* [15]. Likewise, Loehfelm and colleagues determined that the cell surface biofilm associated protein, Bap, contributes to the stabilization of mature biofilms on certain abiotic surfaces [16]. While the identification of these genetic components has increased the understanding of *A. baumannii* biofilm formation, a knowledge gap still exists in understanding the complex process(es) of surface colonization and biofilm formation. Characterizing the molecular components that govern the organism's ability to colonize and persist on abiotic surfaces may lead to novel infection control strategies that eliminate *A. baumannii* colonization and transmission.

In the current study, we set out to expand the characterization of the molecular components that mediate *A. baumannii*'s ability to colonize inanimate surfaces. A transposon mutant library of *A. baumannii* strain 98-37-09 [17], a clinical isolate that displays a high propensity to form biofilms on abiotic surfaces, was screened for members with reduced polystyrene binding. In comparison to the parental strain, one transposon insertion mutant harboring a disruption in the coding region of a ribonuclease T2-family protein (ATCC17978 locus A1S_3026) demonstrated a striking reduction in colonizing polystyrene, polyvinyl chloride endotracheal tubes, glass, and stainless steel surfaces. Microarray analyses revealed that RNase T2 mutation leads to decreased expression of several genes involved in pili formation and motility, commonly associated with biofilm formation in other bacteria including the closely related pathogen *Pseudomonas aeruginosa* (Reviewed in [18]). Complementation restored the mutant strain's ability to colonize abiotic surfaces and led to a partial restoration of cell surface motility phenotype. Taken together, our data suggest that *A. baumannii* RNase T2 family protein regulates surface binding, biofilm formation, and cell motility and thus may represent a target for antimicrobial development.

## Materials and Methods

### Strains and plasmids used in this study

Bacterial strains and plasmids used in this study are listed in Table 1. All strains were grown in either Lysogenic Broth (LB; Becton Dickinson, Franklin Lakes, New Jersey) or Tryptic Soy Broth (TSB; Becton Dickinson). Where indicated, medium was supplemented with kanamycin (50 µg ml^−1^; MP Biomedicals, Solon, OH), ampicillin (50 µg ml^−1^; Thermo Fisher, Waltham, MA), or tetracycline (10 µg ml^−1^; Thermo Fisher). Plasmid pACJ02 was constructed by using primers 3026COMP-F: 5′-CTGCAGCCTTTACAAATAGATTCAATGTATGGT-3′ and 3026COMP-R: 5′-CTGCAGCGTTAAGTGAAATGAACACCAATATT-3′ to PCR amplify the *A. baumannii* strain 98-37-09 RNase T2 family gene and 500 base pair flanking sequences (*Pst*I restriction enzyme sites underlined). The resulting PCR product was cloned into the Invitrogen pCR®II-TOPO® vector and transformed into *Escherichia coli* strain OneShot INVαF' for propagation (Invitrogen; Carlsbad, CA). The resulting plasmid DNA harboring the PCR product was digested with *Pst*I (New England Biolabs, Beverly, MA) to liberate the 1088 base pair fragment containing the *A. baumannii* RNase T2 family gene with flanking sequences, which was then ligated to *Pst*I digested plasmid pWH1266 to generate plasmid pACJ02. Purified pACJ02 or vector was then electroporated into 98-37-09 or ACJ7 cells. Transformants containing the complementation plasmid, pACJ02, or pWH1266 (vector) were selected by growth on LB agar supplemented with tetracycline. The University of Nebraska Medical Center Sequencing Core Facility confirmed the integrity of the plasmid sequences.

**Table 1:** Bacterial strains and plasmids used in this study.

***A. baumannii*** ** Strain**	**Relevant Genotype or Phenotype** 1	**Source or Reference** 2
ATCC 17978	Cerebrospinal fluid	ATCC
98-37-09	Cerebrospinal fluid	[17]
ACJ7	EZ-Tn5::A1S_3026 derivative of 98-37-09	This study
***E. coli*** ** strain**	**Relevant Genotype or Phenotype**	**Source or Reference**
INVαF′	Competent cells	Invitrogen
BL21 (DE3)	Competent cells	Invitrogen
**Plasmids**	**Relevant Genotype**	**Source or Reference**
pCR®II-TOPO®	Amp^r^ Kan^r^	Invitrogen
pWH1266	Amp^r^ Tet^r^	[34]
pET30-LIC	Kan^r^	Novagen
pACJ02	pWH1266/A1S_3026, Tet^r^	This study

1 Ampicillin (Amp); Kanamycin (Kan); Tetracycline (Tet), Resistant (r).

2 American Type Culture Collection (ATCC).

### *A. baumannii* Growth Curves

Overnight cultures of *A. baumannii* strains 98-37-09 and ACJ7 were used to inoculate (1∶100 dilution) culture flasks containing 25 ml of fresh LB medium at a volume-to-flask ratio of 1∶5 and were cultured at 37°C and 225 rpm for 48 h. One milliliter samples were taken at one hour intervals for the first 8 h and then every 12 h for the remainder of the study. Each sample was serially diluted in sterile phosphate-buffered saline (PBS; 137 mM NaCl, 2.7 mM KCl, 100 mM Na_2_HPO_4_, 2 mM KH_2_HPO_4_) and plated on LB agar to enumerate colony-forming units (CFUs) per milliliter. Statistically significant differences in growth rates between wild type and mutant strains were determined by Student's *t* test (*p*<0.05).

### Polystyrene Adherence High Throughput Screen

The *A. baumannii* 98-37-09 transposon mutant library used in these studies has been previously described [17]. Briefly, the library was created using the EZ-Tn5™ <R6Kγ*ori*/KAN-2> Tnp transposome system, following the manufacturer's recommendations for prokaryotic cell transposition (Epicentre Biotechnologies, Madison, WI). In total, the library consists of 6,000 transposon-mutants arrayed in individual wells of 96-well round-bottom plates (Corning, Lowell, MA). To screen for library members with a polystyrene colonization defect, members were transferred to fresh 96-well round-bottom microtiter plates containing 150 µl of TSB supplemented with kanamycin and grown overnight. Approximately 1×10^7^ cells of each test strain were subsequently transferred to new 96-well flat-bottom plates (Becton Dickinson) containing 200 µl TSB. After 48 h incubation at 37°C, cells and media were removed and plates were washed 3 times with PBS (137 mM NaCl, 2.7 mM KCl, 100 mM Na_2_HPO_4_, 2 mM KH_2_HPO_4_). Adherent cells were fixed with 100% ethanol for 15 min. Plates were washed again 3 times with PBS and stained with crystal violet (0.41% W/V; Thermo Fisher) for 1 min. For controls, each microtiter plate contained a non-adherent *A. baumannii* strain (ATCC 17978) in well A1 and wild type 98-37-09 in well A2. A loss of biofilm formation was qualitatively determined by visual inspection of the plates; a complete lack of crystal violet staining in a well was considered a “hit”. All hits were confirmed by secondary quantitative assays, as described below.

### Endotracheal Tube Adherence Assay

*A. baumannii* strains were cultured overnight in LB medium and then subcultured (1∶100) in 100 ml LB medium at a volume-to-flask ratio of 1∶5. Sterile endotracheal tubes (Mallinckrodt, Saint Louis, MO) were inserted into flasks so that half the tube was submerged in the culture medium. Flasks were incubated at 37°C or 25°C for 48 h, endotracheal tubes were removed, washed, fixed, and stained, as described above. To quantify crystal violet staining of adherent cells, tubes were washed 3 times with H_2_O, stain was suspended in 30% glacial acetic acid, vigorously vortexed for 15–20 seconds, and the OD_600_ was measured for each sample. For assays using ACJ7 and ACJ7 complement strains, bacteria were grown in LB supplemented with kanamycin and kanamycin/tetracycline, respectively. Statistically significant differences in adherence were calculated by Student *t*-test and ANOVA for two-strain and three-strain comparisons, respectively (*p*≤0.05).

### Polystyrene, Glass & Stainless Steel Adherence Assay

*A. baumannii* strains were cultured overnight in LB medium and then subcultured (1∶100) in LB medium in 24-well flat-bottom plates or 5 ml polystyrene round-bottom tubes (Becton Dickinson). For glass and stainless steel assays, a glass cover slip or a stainless steel disk was half-submerged in each well of the plate, respectively, and plates were incubated at 37°C or 25°C for 48 h. After 48 h incubation, wells, glass, or stainless steel disks were washed, fixed, and stained, as described above. For assays using ACJ7 and ACJ7 complement strains, bacteria were grown in LB with kanamycin or kanamycin/tetracycline, respectively. Statistically significant differences in adherence were calculated by Student *t*-test and ANOVA for two-strain and three-strain comparisons, respectively (*p*≤0.05).

### Inverse polymerase chain reaction

Inverse PCR was used to identify the transposon insertion site of *A. baumannii* strain ACJ7, as previously described [17]. Briefly, 2 µg of ACJ7 genomic DNA was purified using DNeasy® Blood and Tissue Kit following the manufacturer's recommendations (Qiagen, Valencia, CA), and then digested by restriction enzyme *Afe*I (10 U; New England Biolabs) at 37°C for 1 hr. Restriction fragments were circularized by ligation using 1.5 U of T4 DNA ligase (Invitrogen) at 16°C for 16 h and were subjected to inverse PCR using Platinum® PCR Supermix High Fidelity (Invitrogen) and transposon-specific primers (forward: 5′-ACCTACAACAAAGCTCTCATCAACC-3′; and reverse: 5′-CTACCCTGTGGAACACCTACATCT-3′) supplied in the EZ-Tn5™ <R6Kγ*ori*/KAN-2> Tnp transposome kit (Epicentre Biotechnologies). PCR products were electrophoresed in a 1% UltraPure™ Agarose gel (Invitrogen), purified using a QIAquick® Gel Extraction Kit (Qiagen), ligated into pCR®II-TOPO® and transformed into *E. coli* One Shot® INVαF' cells, following the manufacturer's recommendations for Dual Promoter TA Cloning (Invitrogen). Following propagation, plasmid DNA was purified using QIAprep® Spin Miniprep Kits (Qiagen) and sequenced at the University of Nebraska Medical Center Sequencing Core Facility using vector specific primers (forward: 5′ -GTAAAACGACGGCCAG-3′; reverse: 5′ -CAGGAAACAGCTATGAC-3′).

### Transcriptional profiling

Total bacterial RNA isolation and GeneChip® analysis were performed as previously described [19], [20]. In brief, planktonic bacterial cultures grown in LB medium for 48 h (OD_600 nm_ = 2.2) were centrifuged at 2000 *g* at 4°C for 10 min; each strain was tested in duplicate. Cell pellets were washed in TE buffer (10 mM Tris 10 mM EDTA, pH 7.5), mechanically disrupted in a FP120 shaker (Thermo Scientific), and cellular debris were collected by centrifugation at 16,000 *g* at 4°C for 10 min. The resulting supernatants were used for RNA isolation using Qiagen RNeasy® Mini columns, following the manufacturer's recommendations. For GeneChip® analysis, *A. baumannii* PMDACBA1 microarrays, which are based on the genomic sequence of *A. baumannii* strain ATCC 17978 and additional *A. baumannii* GenBank entries were used, as previously described [19], [20]. These microarrays represent a total of 3,731 predicted *A. baumannii* ORFs and 3,892 ATCC 17978 intergenic regions ≥50 base pairs in length. The GeneChip® data for biological replicates were normalized, averaged, and analyzed with GeneSpring GX 7.3 Analysis Platform software (Agilent Technologies, Redwood City, CA). Genes that exhibited ≥ twofold changes in transcript titer in wild type as compared to those in ACJ7, were determined to be “present” by Affymetrix algorithms during the induced condition, and demonstrated a significant difference in signal (*t*-test *p* value cut-off of ≤0.05) were considered to be differentially expressed. All Genechip data have been deposited in the National Center for Biotechnology Information (NCBI) Gene Expression Omnibus (GEO) microarray repository under accession number GSE52002. Quantitative RT-PCR was performed to verify the microarray-determined profiles of three selected genes, A1S_1510 using primers 5′ CGTACACCTTTTCAAATCAATTTAACG and 5′ GCATAATAATTAAGATCGGCATTCC; A1S_2813 using primers 5′ CTCAAAGTTGGGTCAAGGGATTGGC and 5′ GCAATATTCTTTTAGGTTTTCCTCTAAGTC; and A1S_3194 using primers 5′ GCACAAATCAGAACCTTGATCAGC and 5′ GCTTCAAGATTACGGAGTAGTTCG, as previously described [20].

### Surface Motility assay

Bacterial motility assays were performed as previously described by Mussi and colleagues [21], but with minor modifications. Briefly, motility plates were made with 0.3% agar and LB media without addition of exogenous sodium chloride and were made fresh each time the assay was performed. Three microliters of the indicated culture (grown to an OD_600 nm_ of 0.4) were deposited to the plate's surface and plates were incubated at 37°C for 16 h. The motility of each strain was determined by measuring the halo of growth around the inoculation site. The Wilcoxon signed rank test was used to determine whether there were statistically significant (*p*<0.05) differences between strains tested.

## Results

### Polystyrene adherence

**Figure 1:**
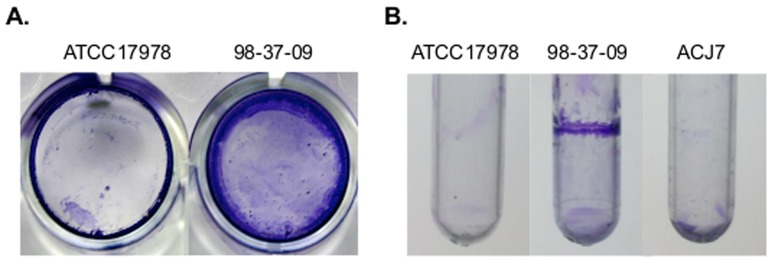
Polystyrene colonization. **Panel A.** Representative images of *A. baumannii* strains ATCC17978 and 98-37-09 colonization of 24 well polystyrene microtiter plates as visualized by crystal violet staining after 48 h incubation at 37°C. **Panel B.** Representative images of *A. baumannii* strains ATCC17978, ACJ7, and 98-37-09 colonization of polystyrene tubes after 48 h incubation.

Colonization of abiotic hospital surfaces by *Acinetobacter baumannii* is thought to provide a reservoir for transmission of the organism to susceptible patients. To identify *A. baumannii* genetic determinants that augment its ability to colonize inanimate surfaces, we initially collected clinical isolates representing 12 genetically distinct lineages and assessed representatives of each lineage for their ability to bind polystyrene 24-well microtiter plates. As shown in Figure 1A., following 48 h incubation, washing and crystal violet staining to qualitatively measure bacterial colonization, the laboratory strain ATCC 17978 exhibited limited ability to adhere to and/or persist on polystyrene surfaces, whereas clinical isolates such as *A. baumannii* strain 98-37-09 exhibited a pronounced ability to bind polystyrene. We reasoned that strains such as 98-37-09, which exhibit a robust biofilm phenotype, harbor unique factors (or unique expression properties) that augment their ability to colonize and/or survive on abiotic surfaces.

### An RNase T2 family protein mediates *A. baumannii* adherence to polystyrene surfaces

To identify *A. baumannii* genetic determinants that contribute to the organism's ability to colonize abiotic surfaces, a 98-37-09 transposon mutant library was screened for members with a reduced polystyrene colonization phenotype. A total of 6,000 *A. baumannii* 98-37-09 transposon mutants were inoculated in individual wells of polystyrene plates and incubated at 37°C for 48 h. Wells were then aspirated, washed, and adherent cells were stained with crystal violet and visually inspected to qualitatively identify library members with a putative colonization deficiency. One mutant, ACJ7, exhibited a marked reduction in polystyrene surface colonization in comparison to wild type cells (data not shown). As a secondary means of verifying those results, we monitored the adherence properties of wild type and ACJ7 cells when cultured in 5 ml polystyrene round bottom tubes. . As shown in Figure 1B., ACJ7 cells exhibited a dramatic reduction in polystyrene surface colonization predominantly at the liquid-air interface in comparison to wild type 98-37-09 cells, whereas the adherence deficient strain, ATCC 17978, demonstrated limited adherence. Growth curve comparisons indicated that the observed differences in adherence were not likely attributable to differences in growth rates between strains (Figure S1), suggesting that ACJ7 harbors a transposon-mediated mutation in a gene that is required for *A. baumannii* adherence and/or colonization of polystyrene surfaces. Accordingly, the transposon insertion site for strain ACJ7 was determined to be located between amino acids 41 and 42 of the predicted 220 amino acid open reading frame of an *A. baumannii* RNase T2 family protein (ATCC 17978 locus A1S_3026) by inverse PCR.

**Figure 2:**
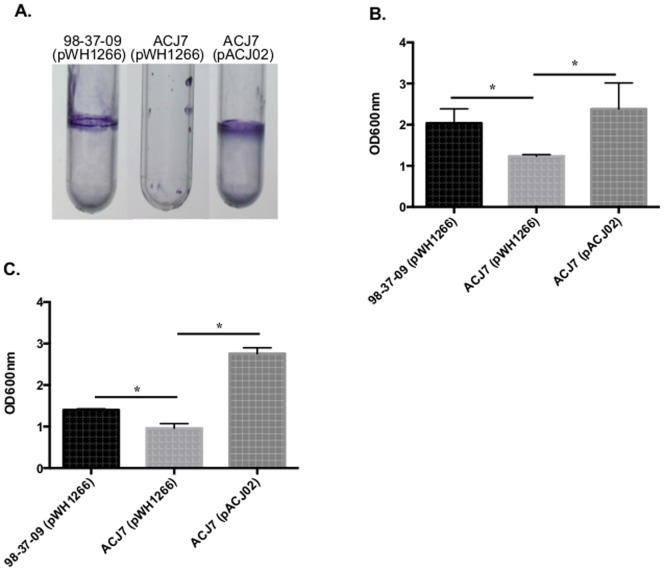
Adherence characteristics of RNase T2 complemented cells. **Panel A.** Representative images of *A. baumannii* 98-37-09 pWH1266 (vector control), ACJ7 pWH1266, and ACJ7 pWH1266 (RNase T2 family gene in plasmid pWH1266) colonization of polystyrene tubes after 48 h incubation at 37°C. **Panel B.** Graphed are the corresponding average crystal violet staining measures of replicate (n = 3) polystyrene colonization experiments, as measured by optical density of resuspended stain in glacial acetic acid. **Panel C.** Graphed are the crystal violet staining measures of *A. baumannii* strains 98-37-09 pWH1266, ACJ7 pWH1266, and ACJ7 pACJ02 following 48 h incubation in in polystyrene plates at 25°C (n = 6/strain). Standard deviations are shown. Asterisks indicate statistically significant differences between wild type, ACJ7, and complement strain adherence as determined by two-way Analysis of Variance (*p*<0.05).

Complementation was used to distinguish whether the reduced polystyrene adherence properties of ACJ7 cells were due to disruption of the strain's RNase T2 family locus, as opposed to other unappreciated features of the strain. As shown in Figures 2A. and 2B., the colonization phenotype of ACJ7 cells could be restored to that of wild type levels by complementation with plasmid pACJ02 harboring a wild type copy of the RNase T2 family gene, whereas ACJ7 cells containing vector alone (pWH1266) did not restore adherence. Moreover, quantitative RT-PCR measures revealed no significant difference in mRNA titers of A1S_3026 flanking genes (A1S_3025 and A1S_3027) between wild type and ACJ7 cells (data not shown). Taken together these results suggest that the disruption of the A1S_3026 RNase T2 family locus, as opposed to transposon-associated polar effects, directly limits the polystyrene colonization properties of ACJ7 cells.

While initial adherence assays were performed at 37°C to mimic *A. baumannii* colonization of indwelling devices, we also investigated whether A1S_3026 mutation also affected the organism's ability to colonize polystyrene surfaces at room temperature to better represent environmental conditions and surfaces within the health care setting. Accordingly, adherence assays were repeated at 25°C with *A. baumannii* strains 98-37-09, ACJ7, and A1S_3026 complemented ACJ7 cells. As shown in Figure 2C., colonization of polystyrene microtiter plates was significantly reduced for ACJ7 in comparison to wild type cells (*p*<0.01). Further, colonization by the complementation strain exceeded that of wild type cells; presumably, this increase is attributable to the high copy number of the complementation plasmid and corresponding overexpression of the RNase T2 family protein. Taken together, these results suggested that a product of the A1S_3026 RNase T2 family protein locus contributes to *A. baumannii* adherence and/or colonization of polystyrene surfaces at temperatures expected within the health care environment and host.

### Characterizing the adherence properties of RNase T2 family mutant cells

To distinguish whether the adherence defect of ACJ7 cells was specific to polystyrene surfaces, studies were expanded to evaluate the adherence properties of wild type, ACJ7 and complemented cells on polypropylene, glass, and stainless steel surfaces at 25°C and/or 37°C.

**Figure 3:**
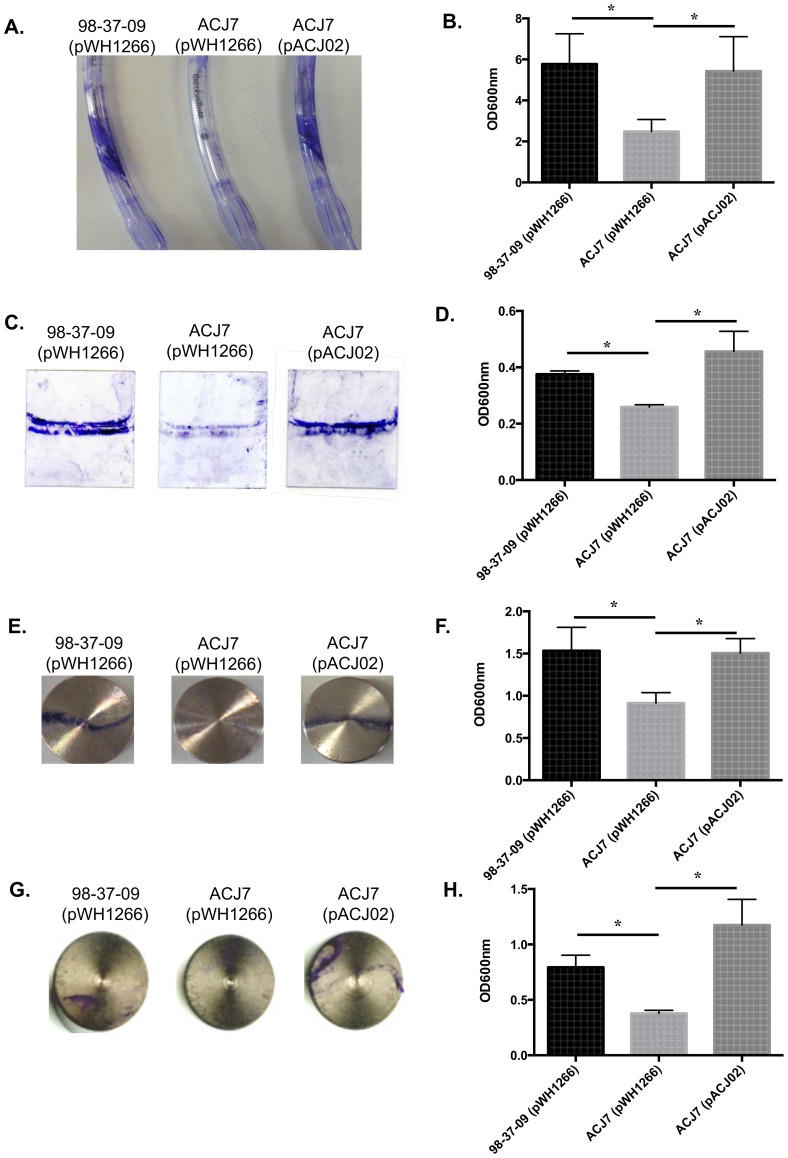
Surface colonization properties of *A. baumannii* strains. Shown are images and corresponding quantified measures of solubilized crystal violet stained *A. baumannii* wildtype, ACJ7, and RNase T2 family complemented cells to polypropylene endotracheal tubes at 37°C (**Panels A** and **B**; n = 3/group), glass cover slips (25°C; **Panels C** and **D**; n = 3/group), and stainless steel disks (25°C; **Panels E** and **F**; and 37°C; **Panels G** and **H**; n = 5/group for these disks). Asterisks indicate statistically significant differences between wildtype, ACJ7, and complement strain adherence as determined by two-way Analysis of Variance (*p*<0.05). Each data set is representative of at least two independent experiments.

As stated above, one of the most severe forms of *A. baumannii*-associated disease is ventilator-associated pneumonia; one could imagine that colonization of ventilator intubation tubing provides a reservoir for *A. baumannii* dissemination to the lung epithelia and subsequent pathogenesis. Thus, we assessed whether the A1S_3026 mutation within ACJ7 cells also affected the organism's ability to adhere to polypropylene endotracheal intubation tubing at 37°C. As shown in Figures 3A and 3B., wild type 98-37-09 cells strongly colonized the endotracheal tube, whereas adherence was nearly eliminated for ACJ7 cells or ACJ7 cells containing plasmid pWH1266 (vector). Conversely, ACJ7 cells harboring a plasmid borne copy of A1S_3026 (pACJ02) displayed increased polypropylene tube colonization in comparison to wild type cells, suggesting that the A1S_3026 RNase T2 family protein locus is also required for *A. baumannii* colonization of polypropylene surfaces.

The observation that the A1S_3026 locus is required for wild type levels of *A. baumannii* polystyrene and polypropylene colonization led us to assess whether it also affects the organism's colonization of other abiotic surfaces commonly found in the hospital setting. Accordingly, the colonization properties of wild type, ACJ7, and A1S_3026 complemented ACJ7 strains to glass and stainless steel were measured using a modified 24-well plate assay. Because glass is present in the patient-care environment whereas steel is present in both the environment and implanted medical devices, adherence to glass cover slips was measured at 25°C, while adherence to stainless steel was assayed at 25°C and 37°C. The RNase T2 family mutant, ACJ7, exhibited a slight, but statistically significant (*p*<0.05), reduction in adherence to glass cover slips in comparison to wild type and complemented strains (Figures 3C. and 3D.). Similarly, ACJ7 cells displayed a reduction in colonization of stainless steel disks; quantitative analysis revealed a 40% reduction in ACJ7 adherence at 25°C (Figures 3E. and 3F.) and 52% reduced adherence at 37°C (Figures 3G. and 3H.), as compared to wild type and A1S_3026 complemented ACJ7 cells. Further, a comparison of the stainless steel adherence properties of the wild type strain at 25°C and 37°C suggests that *A. baumannii* may have a greater propensity to colonize steel at room temperature.

### RNase T2-dependent changes in gene expression

The *A. baumannii* A1S_3026 locus is predicted to code for a Ribonuclease T2 family protein. Proteins within this family have been shown to regulate an array of biological processes, including nutrient scavenging and stress adaptation, in other organisms (reviewed in [22]). Thus, we used microarrays to investigate whether the putative *A. baumannii* RNase T2 family protein affects the expression properties of genes associated with bacterial adhesion and/or biofilm formation. To do so, total cellular RNA was extracted from exponential phase wild type, ACJ7, and complemented cells and was subjected to microarray expression analysis. As summarized in Table 2, a total of 29 genes displayed ≥2-fold (*p*<0.05) decrease in transcript titers in ACJ7 as compared to wild type and complemented cells, suggesting that the corresponding decrease in expression of these genes may contribute to the adherence defect of RNase T2 mutant cells. As expected, transcript titers of the A1S_3026 RNase T2 family mRNA species were increased 6.6-fold and 155-fold in wild type and complemented cells in comparison to the mutant strain, verifying that the microarray study was appropriate to identify genes that are regulated in an RNase T2 family-dependent manner. Among the remaining 28 genes displaying increased titers in both wild type and RNase T2 family complemented cells, we noted that half (14 genes; 50%) represented genes predicted to be adjacent to one another, suggesting that they are members of four operons. These include ATCC17879 loci: A1S_0328 and A1S_0329; A1S_1509 and A1S_1510; A1S_2811 to A1S_2815; and A1S_3191 to A1S_3195, which collectively displayed 3.6 (±1.2)-fold and 10.8 (±5.5)-fold increases in transcript titers within wild type and RNase T2 family complemented cells, relative to mutant cells. Moreover, all of the genes located within each of these putative operons, are predicted to code for proteins associated with pili assembly and cell motility, features that are associated with biofilm formation in other bacterial species [18], [23]–[26]. To verify our microarray results, quantitative RT-PCR (qRT-PCR) was used to measure the mRNA levels of members of three of the aforementioned putative operons, which could ostensibly be associated with *A. baumannii* surface colonization, within wild type, ACJ7 and complemented cells. qRT-PCR results revealed that the transcript levels of A1S_1510, which is predicted to code for a fimbrial protein were increased 4.7 and 7.4 fold within wild type and complemented cells, respectively, in comparison to ACJ7. Likewise, A1S_2813 (twitching motility protein) mRNA levels increased 7.4 and 8.9 fold, whereas A1S_3194, encoding the *pilN*/*comN* component of pilus/fimbrial assembly increased increased 2.0 and 3.1 fold, respectively, for wild type and complemented cells, in comparison to ACJ7 cells.

**Table 2:** Genes down-regulated in RNase T2 mutant cells.

	Fold Increase within				
Array ID	98-37-09	ACJ7 (pACJ02)	Locus 1	Gene Name	Description
gi-126640194	2.4	10.8	A1S_0087		Short-chain dehydrogenase/reductase SDR
**gi-126640410**	**2.5**	**4.4**	**A1S_0328**		**type 4 fimbrial assembly protein**
**gi-126640411**	**2.6**	**7.3**	**A1S_0329**		**type 4 fimbrial biogenesis protein**
gi-126640498	3.2	8.7	A1S_0425		hypothetical protein
gi-126640604	2.1	2	A1S_0533		hypothetical protein
gi-126640619	4.5	3.2	A1S_0548		putative transcriptional regulator (TetR family)
gi-126641234	2.7	4.6	A1S_1185		ATP-dependent protease Hsp 100
gi-126641304	3	7	A1S_1258		hypothetical protein
gi-126641365	3.7	2.2	A1S_1319		hypothetical protein
gi-126641522	3.6	6.6	A1S_1476		hypothetical protein
**gi-126641554**	**4**	**3.9**	**A1S_1509**		**pili assembly chaperone**
**gi-126641555**	**5.9**	**6.6**	**A1S_1510**		**Fimbrial protein**
gi-126642117	2.6	8.1	A1S_2072	*usp*	putative universal stress protein family
gi-126642696	2	3	A1S_2662		putative hydrolase
gi-126642817	2.2	2	A1S_2787		hypothetical protein
**gi-126642835**	**2.2**	**10.9**	**A1S_2811**		**chemotactic signal transduction system component**
**gi-126642836**	**3.7**	**13.4**	**A1S_2812**	***pilJ***	**Type IV pili methyl-accepting chemotaxis protein**
**gi-126642837**	**5.4**	**23.2**	**A1S_2813**		**twitching motility protein**
**gi-126642838**	**2.6**	**6.5**	**A1S_2814**		**twitching motility protein**
**gi-126642839**	**2.7**	**6.7**	**A1S_2815**		**twitching motility protein**
gi-126643041	6.6	155	A1S_3026		RNase T2
**gi-126643183**	**2.1**	**7.9**	**A1S_3168**	***pilW***	**pilus assembly protein**
**gi-126643202**	**3.4**	**14.2**	**A1S_3191**	***comQ***	**type IV pilus secretin**
**gi-126643203**	**2.9**	**10.4**	**A1S_3192**	***pilP/comL***	**pilus assembly protein**
**gi-126643204**	**4.1**	**15.7**	**A1S_3193**	***pilO/comO***	**pilus assembly protein**
**gi-126643205**	**5**	**16.9**	**A1S_3194**	***pilN/comN***	**fibrial assembly protein**
**gi-126643206**	**3.5**	**11.9**	**A1S_3195**	***comM***	**competence protein A**
gi-126643244	2.8	4	A1S_3236		hypothetical protein
gi-126643330	2.3	10.7	A1S_3323		putative flavoprotein

1 ATCC17978 locus tag; adjacent loci are highlighted in grey; loci associated with pili formation and motility are bolded.

**Figure 4:**
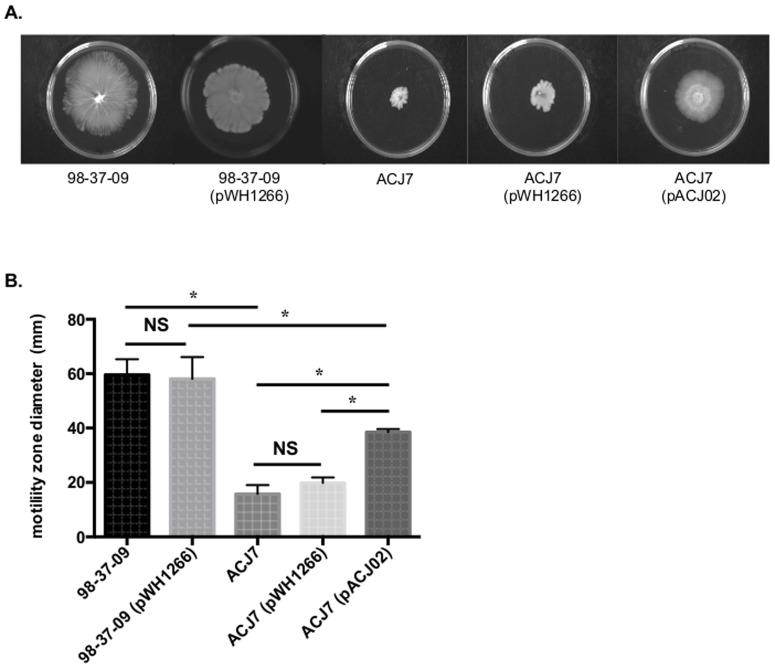
Motility of *A. baumannii* strains. **Panel A.** Images displaying the motility properties of *A. baumannii* strains 98-37-09, 98-37-09 (pWH1266; vector), ACJ7, ACJ7 (pWH1266), and ACJ7 (pACJ02; RNase T2 complementation plasmid). **Panel B.** For each strain, the diameter across the largest portion of the resulting cell motility halo was measured in millimeters, averaged (n = 3) and plotted; the Wilcoxon rank sum test (NS: not significant, i.e. *p*≥0.05, or significant at *p*<0.05) are shown above each pair of strain-specific data.

To further verify our microarray data and explore the possibility that RNase T2 family mutant cells down regulate motility-associated genes, surface motility assays were performed on wild type, RNase T2 family mutant, and complemented cells. As shown in Figure 4A. and 4B., we observed that the 98-37-09 wild type strain exhibit robust surface motility phenotype that is minimally affected by the presence of the pWH1266 vector. In contrast, the ACJ7 strain exhibited a markedly decrease in cellular surface motility that was not measurably affected by the presence of the pWH1266 vector alone but was complemented to statistically significant levels by the presence of the pACJ02 plasmid. Thus, our gene expression data correlates with cellular surface motility activity of the RNase T2 family mutant.

## Discussion

Our understanding of genes and their cognate proteins involved in *A. baumannii* surface attachment, colonization, and biofilm formation is limited and remains an urgent research priority due to the immediate health threat posed by this bacteria. To this end, we screened a collection of transposon insertion mutants of the *A. baumannii* 98-37-09 biofilm-producing strain. Intriguingly, we identified an *A. baumannii* RNase T2 family gene as a regulator of abiotic surface colonization and cellular motility phenotypes of this strain. Whereas previous studies of genes involved in surface attachment and/or biofilm formation identified those encoding structural proteins, our data suggest that a regulatory molecule with high degree of homology to members of the T2 ribonuclease family of proteins positively modulates such phenotypes [22].

Interestingly, ACJ7 was the only member of the mutant library screened in this study that demonstrated decreased polystyrene colonization. This was initially unexpected; we anticipated that screening would also identify mutants with transposon insertions in previously described adherence-related genes (i.e., *ompA* or the *csu* operon). There are at least two possibilities as to why these mutants were not found during our phenotypic screen. First, our estimated probability of transposon mutagenesis disrupting a gene within the entire 98-37-09 genome is ∼78%. Thus, it is theoretically possible that mutations affecting some of the previously characterized biofilm-associated loci are not represented in the transposon mutant library. Second, during our initial phenotypic screen, we sought a complete loss of adherence to polystyrene among our existing panel of mutant strains. The stringency of our screening assay may have reduced the probability of our identifying mutants with qualitatively reduced polystyrene adherence.

Our identification of RNase T2 family protein as a modulator of *A. baumannii* surface colonization and cellular motility was unexpected since previously studied RNase T2 family orthologues had not been associated with either of these processes. Prototypical members of this family, almost all of which have been characterized from eukaryotic organisms, possess non-specific endoribonuclease activity that involve two histidine active site residues during the formation of the 2′, 3′-cyclic phosphate intermediate; very little is known about prokaryotic RNase T2 family members [22]. In eukaryotes RNase T2 proteins are primarily localized within the intracellular compartments, but they may also be secreted into the extracellular environment for RNA degradation and scavenging [22]. Our structure-function analysis of the *A. baumannii* A1S_3026 RNase T2 family protein revealed no obvious secretory motifs and showed that the predicted molecular mass and amino acid residues comprising the putative active site within the Conserved Amino acid Sequence (CAS) I/II domains of the protein are similar to those of other RNase T2 proteins. However, we found that the *A. baumannii* A1S_3026 RNase T2 family protein lacked the two histidines within the CAS domains and did not observe detectable ribonuclease activity of purified RNase T2 *in vitro* (data not shown). We cannot formally exclude potential limitations in our ribonuclease assays to detect RNase T2 activity, and it is also possible that the protein functions as a ribonuclease in the presence of unidentified co-factor(s). However, in our BLAST searches and literature reviews, we do note that *Chromobacterium* RNase T2 possesses only one of two histidines and that three of eight *Tetrahymena* RNase T2 orthologues lack both histidines within the CAS domains [27], [28]. Also, there are precedence for eukaryotic members of the ribonuclease T2 protein family to possess ribonuclease-independent regulatory functions, such as growth inhibition in yeast and putative tumor suppressor activities of *Aspergillus* and human orthologues [29]–[31]. Thus, the *A. baumannii* putative RNase T2 family protein identified in this study may regulate bacterial surface attachment in a manner that is not associated with RNase activity.

Our complementation and gene expression data suggest that the RNase T2 family protein modulates surface colonization and cell motility in the biofilm-forming *A. baumannii* 98-37-09 strain. In other bacteria, such as *Bacillus subtilis*, *Pseudomonas aeruginosa*, *Neisseria meningitides*, and *Myxococcus xanthus*, cellular motility is inherently and functionally linked to biofilm formation [18], [23], [24], [26], [32]. Our data suggests that a similar relationship between surface motility and biofilm formation exists in *A. baumannii*, and that the RNase T2 family protein identified here regulates both processes. Indeed, gene expression studies revealed that loss of RNase T2 family function is associated with decreased expression of several genes involved in cellular motility as well as others (e.g. pili formation) involved in biofilm formation. Such results are in accord with other bacterial ribonucleases that affect the expression of target genes; one such example is the *Enterococcus faecalis* RNase J2 that is required for pilin gene expression and biofilm formation on polystyrene surfaces [33]. PHYRE analysis of hypothetical genes that are expressed in an RNase T2 dependent manner, including: A1S_0425 (alpha helical protein of unknown function), A1S_1258 (transport protein), A1S_1319 (limited homology to RNA binding/nucleotide hydrolase), A1S_1476 (tautomerase/hydrolase), A1S_2787 (limited homology to *Bartonella* cell invasion associated protein) and A1S_3236 (homology to tRNA transferase/hydrolase) did not provide additional insight regarding how A1S_3026 modulates abiotic surface colonization. Thus, we surmise that the RNase T2 family protein-associated biofilm formation phenotype is predominately mediated by the protein's regulatory effects on motility and pili assembly proteins. None the less, the incomplete rescue of cell surface motility phenotype suggest that the protein exerts its phenotypic effects via multiple target genes, including but not limited to those affecting surface motility. Interestingly, during the course of our investigations, Harding and colleagues found that the *A. baumannii* strain M2 is capable of producing type IV pili which is, in turn, responsible for a second type of motility (twitching motility) and natural transformation [35]. It is intriguing to consider that the RNase T2 family protein identified here may play a role in those biological processes. The precise biochemical roles of *A. baumannii* RNase T2 family protein to regulate gene expression, surface attachment, biofilm formation, and cell motility, remain as targets for future studies as well as antibacterial drug development.

## Supporting Information

Figure S1 **The growth characteristics of** ***A. baumannii*** **strains 98-37-09 (boxes) and ACJ7 (diamonds) in LB medium; standard deviations shown (n = 3).**
(TIF)
