# Supplementary figures and images for: An Ribonuclease T2 Family Protein Modulates Acinetobacter baumannii Abiotic Surface Colonization
Source: PLoS One. 2014 Jan 28;9(1):e85729. doi: 10.1371/journal.pone.0085729 (PMC3904860; doi:10.1371/journal.pone.0085729)

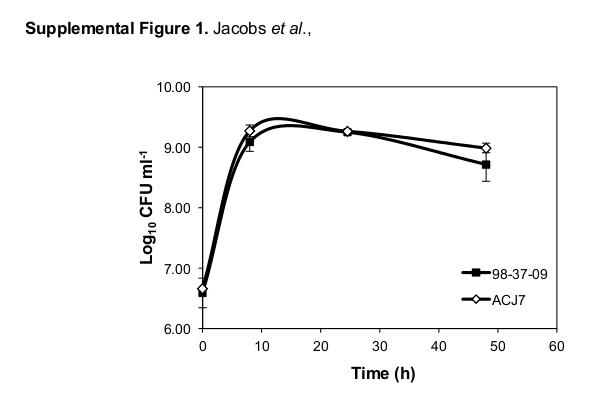

Supplement: Figure S1 — The growth characteristics of A. baumannii strains 98-37-09 (boxes) and ACJ7 (diamonds) in LB medium; standard deviations shown (n = 3). (TIF) [file pone.0085729.s001.tif]
